# Validation of the Physician Teaching Motivation Questionnaire (PTMQ)

**DOI:** 10.1186/s12909-015-0448-5

**Published:** 2015-10-02

**Authors:** Christoph Dybowski, Sigrid Harendza

**Affiliations:** Department of Internal Medicine, University Medical Center Hamburg-Eppendorf, III. Medizinische Klinik, Martinistr. 52, D-20246 Hamburg, Germany

**Keywords:** Motivation, Physician, Self-determination theory, Self-efficacy, Teaching, Teaching involvement, Undergraduate medial education

## Abstract

**Background:**

Physicians play a major role as teachers in undergraduate medical education. Studies indicate that different forms and degrees of motivation can influence work performance in general and that teachers’ motivation to teach can influence students’ academic achievements in particular. Therefore, the aim of this study was to develop and to validate an instrument measuring teaching motivations in hospital-based physicians.

**Methods:**

We chose self-determination theory as a theoretical framework for item and scale development. It distinguishes between different dimensions of motivation depending on the amount of self-regulation and autonomy involved and its empirical evidence has been demonstrated in other areas of research. To validate the new instrument (PTMQ = Physician Teaching Motivation Questionnaire), we used data from a sample of 247 physicians from internal medicine and surgery at six German medical faculties. Structural equation modelling was conducted to confirm the factorial structure, correlation analyses and linear regressions were performed to examine concurrent and incremental validity.

**Results:**

Structural equation modelling confirmed a good global fit for the factorial structure of the final instrument (RMSEA = .050, TLI = .957, SRMR = .055, CFI = .966). Cronbach’s alphas indicated good internal consistencies for all scales (α = .75 – .89) except for the identified teaching motivation subscale with an acceptable internal consistency (α = .65). Tests of concurrent validity with global work motivation, perceived teaching competence, perceived teaching involvement and voluntariness of lesson allocation delivered theory-consistent results with slight deviations for some scales. Incremental validity over global work motivation in predicting perceived teaching involvement was also confirmed.

**Conclusions:**

Our results indicate that the PTMQ is a reliable, valid and therefore suitable instrument for assessing physicians’ teaching motivation.

## Background

In its history, education in general and medical education in particular experienced a shift from teacher-centered learning formats - such as lectures - to student-centered forms of learning - such as problem-based-learning [1, 2]. Research in medical education reflects this development with one major focus being on student outcomes and a recent claim for a stronger additional consideration of patient outcomes [3, 4]. Motivational theories with broad empirical support and great influence in the recent decades, such as the social cognitive theory and the self-determination theory, imply that motivation influences task selection, persistence and performance [5, 6]. When students’ learning progress is regarded as an aspect of teaching performance, these theories suggest that not only students’, but also teachers’ motivation might be important to ensure successful student learning. In general, studies from undergraduate education research have demonstrated effects of teacher characteristics on student achievement [7, 8]. More specifically, teachers’ autonomous teaching motivation can also enhance autonomous learning motivation in their students [9–11], and students’ autonomous learning motivation can positively affect academic performance [12].

However, modern medical curricular developments focus predominantly on students’ cognitive and metacognitive regulation than on enhancing motivation [13]. Teachers’ motivations seem to be even more underrepresented in medical education development and research. In a review of 53 papers concerning faculty development initiatives [14], only four mentioned the assessment of teaching motivation or “enthusiasm”, as an outcome variable [15–18]. Furthermore, no validated instrument is currently available to specifically assess physicians’ teaching motivations in undergraduate medical education. In contrast, a review of 68 mostly descriptive articles regarding the characteristics of good clinical teachers in medical education found “demonstrat(ing) enthusiasm for teaching” in 18 articles and as the fifth most frequently mentioned category [19]. Furthermore, we assume in the context of motivation theories that other teacher characteristics described in these studies like “being accessible to students”, “demonstrating commitment to teaching improvement” or “maintaining positive relationships with students” also constitute expressions of teaching motivation.

### Self-determination theory

As a theoretical framework for the construction of our teaching motivation questionnaire, we chose the self-determination theory (SDT). It is a macro-theory postulating a multidimensional view of motivation including its antecedents and its consequences across different life domains and currently represents one of the most recognized motivational theories in psychology [6]. SDT distinguishes between three major types of motivation depending on the level of self-regulation and autonomy, respectively, involved. Firstly, *intrinsic motivation* represents the type of motivation with the highest self-regulation. It gives rise to actions which are done out of pure interest or joy and which are non-instrumental in nature. Secondly, *extrinsic motivation* comprises three different types of motivation, *external regulation*, *introjected regulation*, and *identified regulation*, whose conjoint characteristic is that related actions are not carried out for the action itself but are instrumental in nature. Thirdly, *amotivation* is defined as the absence of motivation. With respect to the second category, e*xternal regulation* represents the least self-regulated subtype of extrinsic motivation and refers to activities solely conducted to obtain rewards or to avoid punishments. *Introjected regulation* refers to activities with which someone does not completely identify and which are regulated by internal pressures, such as feelings of guilt and shame. *Identified regulation* refers to actions based on personal values and beliefs. While *introjected regulation* is accompanied by a feeling of being controlled by internal pressures, *identified regulation* represents voluntary behaviours driven by conviction.

According to SDT, the more self-regulated an action is, the higher the invested efforts as well as the well-being of the acting person will be. In accordance with these theoretical considerations, empirical findings from the field of work psychology show that more self-regulated types of motivation, especially intrinsic motivation, predict better performance, greater commitment, and more job satisfaction [20]. Furthermore, SDT postulates that motivation types are not static, but permeable. *Internalization* describes the process of adopting and integrating extrinsic motifs into one’s own individual set of values and thus, achieving a higher level of self-regulation. According to SDT, environments that provides opportunities for experiencing *autonomy*, *competence* and social *relatedness* can facilitate this process as these three factors constitute basic human needs and are important predictors of motivation [6].

The potential of SDT for improving medical education for students as well as faculty staff was acknowledged by Ten Cate et al. who promote a stronger consideration of its principles in the medical curriculum and in faculty development under consideration of educators’ needs [21]. Therefore, the aim of our study was to develop and validate an instrument to detect physicians’ teaching motivation. As modern concepts of validity refer to “the degree to which evidence and theory support the interpretations of test scores entailed by proposed uses of tests” [22], we wished to develop an instrument as specific as possible for teacher-related situations such as the evaluation of faculty development programs or finding incentives for teaching with the aim to provide high-quality teaching.

## Methods

In classical conceptualizations, validity has been defined as three separate types, content, construct and criterion validity [23]. For this study, we defined validity following modern conceptualizations in which validity is a unitary concept with construct validity as a core, deriving validity evidence from several sources such as assessments of content validity, the response process, the internal structure of the instrument, its relationships to other variables and its consequences [22, 24]. For this study, we focused on a careful development of the items to ensure content validity. The response process was analysed with participants of the target group. The internal structure was assessed with respect to dimensionality and scale reliabilities. The relationship to other variables was assessed in terms of convergent validity, concurrent criterion validity and incremental concurrent validity over global work motivation. The consequences of testing are discussed in the context of the fields of usage for our instrument.

### Development of the PTMQ (Physician Teaching Motivation Questionnaire)

In a first step, we developed items for the intrinsic motivation (12 items), identified regulation (four items), introjected regulation (seven items), external regulation (12 items) and amotivation (five items) subscales. Item development occurred theory-driven based on the definitions of the motivational categories as proposed by SDT [6]. The items for external regulation included three items for career motifs, eight items for social regulation and one item, which did not fit either category but was assumed to constitute an important external motif (“I mostly teach because it belongs to my scope of duties”). As a stem, we used “Please state in how far the following statements with potential reasons to participate in teaching apply for you.” Most items began with “I teach because…” followed by a specific reason that matched the underlying motifs of the subscales. A five-point Likert-scale of agreement was used for the rating of these items from “does not apply at all” (=0) to “fully applies”(=4). A modification of item construction was necessary in several cases. All items for amotivation were formulated starting “I teach although…” (e.g. “I teach although I hardly ever feel like doing it”). While SDT defines amotivation as the absence of motivation and therefore the absence of reasons to do a task, we also formulated items that express a stronger sense of aversion towards teaching. Furthermore, we did not include “I teach, because…” in several items for the intrinsic motivation subscale, when the statement itself already expressed intrinsic motivation, e.g. “I enjoy trying new teaching methods”.

In the next step, we conducted a cognitive debriefing with three physicians working in the Department of Internal Medicine at the University Medical Center Hamburg-Eppendorf to analyse the response process and to further enhance content validity. First, participants filled out the preliminary questionnaire and discussed the comprehensibility of all items. One item for amotivation had to be reformulated. Afterwards, the underlying theoretical framework of the questionnaire was presented and a group discussion was initiated about the items’ adequacy as representations of this framework. Three items for external regulation, which refer to teaching as a work task, were reformulated as participants noted that teaching is part of their implicit or explicit work obligations, so they would have to agree completely which would result in biased values. During the discussion, we agreed with the participants to add “mostly” to these items, e.g. “I teach mostly because teaching is a part of my work tasks.” As a result, all items were found to be comprehensible as well as representative of their theoretical constructs and the domain of work at a university hospital.

SDT also postulates the existence of a motivational category called *integrated regulation* as a subtype of extrinsic motivation that ranges between identified and intrinsic regulation with respect to the level of self-regulation. Several authors of other instruments based on SDT have refused to include this category [11, 25, 26] because of dissatisfying results of factor analyses where integrated regulation lacked statistical discriminability from intrinsic and identified regulation [27, 28]. Therefore, we did not develop items for this category. The item order of the questionnaire was determined using a random number generator in SPSS.

### Further instruments and materials

#### Global work motivation

We assumed that task-specific motivation, in this study teaching motivation, and global motivation reflecting the same domain (i.e. work) should correlate positively. Therefore, corresponding task-specific teaching motivation scales should correlate with global work motivation scales as teaching motivation represents a part of global work motivation. In order to test this, we included the German version of the *Multidimensional Work Motivation Scale* (MWMS; [25]) in our questionnaire. The MWMS is a 19-item instrument also based on SDT and has been validated in seven languages and nine countries. Confirmatory factory analyses showed the same factorial structure across all countries, resulting in the subscales intrinsic, identified, introjected, external motivation and amotivation, with *external motivation* being a higher order factor comprising the subordinate factors *extrinsic social* and *extrinsic material*. Internal consistency is good to excellent in all subscales (α = .70 – .94), except for the subscales *identified* (α = .65) and *introjected* (α = .55) in the German samples.

For further criterion validation, we included two constructs supported by SDT into the questionnaire: teaching self-efficacy as a theoretic predictor of motivation and teaching involvement as a theoretic outcome of motivation. Since a literature search for validated instruments aimed at hospital-based physicians yielded no results, we developed new scales for both constructs.

#### Teaching self-efficacy (TSE)

While SDT defines “perceived competence” to be one of the basic human needs giving rise to autonomous motivation, we decided to employ an instrument measuring “self-efficacy”, which is a central construct in the social cognitive theory referring to “beliefs in one’s capabilities to organize and execute the courses of action required to produce given attainments” [29]. While both constructs aim at the belief in one’s ability to master a certain task, perceived competence in SDT stresses the personal meaningfulness and importance of a task from which a person’s need satisfaction depends on [30]. As we assume that physicians rate their personal importance of teaching very differently and that both constructs share a great overlap, we chose to assess self-efficacy for this validation study. Self-efficacy scales have been developed across a wide range of subjects in psychology including teachers’ self-efficacy [31, 32]. In order to assess TSE, we followed Bandura’s guidelines for the construction of scales measuring self-efficacy [33]. Following Bandura’s recommendation to let participants rate their competences when facing difficult and specific situations, we formulated 16 items that represent typical critical situations regularly faced by medical teachers, e.g. time strain, problems with patients and patient selection, interruptions, short-term allocation of teachers to lessons or demotivated students [34–38]. A five-point Likert-scale of agreement was used for the rating of these items. Included items were, for example: “Even when I feel stressed or when I am in a bad mood, I teach well” and “Even when students seem tired or demotivated I succeed in motivating them with my teaching”. In the sample of this study, the TSE scale showed a good internal consistency (Cronbach’s α = .87).

#### Perceived teaching involvement (PTI)

We defined PTI as the endeavour to utilize personal behavioural and cognitive resources actively to achieve good teaching performance. Following our definition, we constructed 15 statements about engaging behaviourally and/or cognitively in teaching before, during and after a lesson indicating efforts to provide high quality teaching, e.g.: “I try to prepare each lesson carefully” and “It is very important for me to provide good teaching”. Our indicators of PTI where partly taken from literature [19] and partly from our own comprehensive practical experience in medical teaching. A five-point Likert-scale of agreement was used for the rating of these items. In the sample of this study, the PTI scale showed a good internal consistency (Cronbach’s α = .87).

#### Lesson allocation

We further assumed that physicians who voluntarily choose to be involved in teaching show higher values in intrinsic and identified teaching motivation and lower values in external teaching motivation and amotivation in comparison to teachers who were are allocated by coordinators with or without having been asked. Therefore, we asked how allocation to teaching had primarily occurred within the last year. Options for answering were “I applied voluntarily for teaching”, “I was asked whether I would like to take over teaching lessons”, and “I was allocated to teaching lessons without being asked”.

As socio-demographic characteristics, age, gender, occupational position, medical specialty, teaching experience in years, occupational position and status of postdoctoral lecture qualification were gathered. The final questionnaire was distributed in a paper-pencil version to 645 clinical teachers form the departments of internal medicine and surgery who are mostly involved in bedside teaching. At German University Medical Centers, professors, consultants, and all residents are involved in clinical teaching independently of their intended career paths. The Ethics Committee of the Hamburg Chamber of Physicians confirmed the innocuousness of this study and its congruence with the Declaration of Helsinki.

### Data analysis

#### Data preparation

All Likert scales in the following statistical analyses were treated as interval scales [39]. Missing values in the PTMQ, the MWMS as well as the PTI and TSE scales were replaced using the EM-algorithm in SPSS if at least 90 % of answered items in the respective scale were present per participant. When only a smaller percentage of answered items was present in the respective scale, questionnaires were excluded from calculations involving the respective scale.

#### Item selection and factorial validity

To select items and to test the assumed factorial structure of our instrument, we performed structural equation modelling (SEM) in SPSS AMOS® 22 using maximum likelihood estimation based on the covariance matrix of the items. The presupposition of univariate normal distribution of the PTMQ items was tested based on the recommendations of West et al. [40]. None of the items exceeded a skewness >2 (range from −1.297 to 1.357) and a kurtosis >7 (range from -.934 to 3.266). All tested models were recursive without allowing correlated error variances. Intercorrelations of all factors were allowed. In order to determine the best items for each factor, backwards elimination was used; items were eliminated subsequently if they did not meet the following criteria: factor loadings >.05 and low cross-loadings on other factors. To assess global model fit, the Root Mean Square Error of Approximation (RMSEA), the Standardized Root Mean Square Residual (SRMR), the Tucker-Lewis Index (TLI) and the Comparative Fit Index (CFI) were calculated. Recommended cut-off-values for the RMSEA range from <0.05 to <0.08, for the SRMR from <0.05 to <0.08 and for the CFI and the TLI from ≥0.95 to ≥0.80 (most strict recommendations presented first; [41]). For the assessment of local goodness-of-fit, factor loadings and indicator reliabilities (squared factor loadings) were calculated.

#### Scale characteristics

Means, standard deviations, intercorrelations and internal consistencies using Cronbach’s α were calculated for the final scales. Internal consistencies were evaluated using the recommendations according to Kline (α ≥0.9 = excellent; 0.7 ≤α <0.9 = good; 0.6 ≤α <0.7 = acceptable; 0.5 ≤α <0.6 = poor; α <0.5 = unacceptable) [42].

#### Criterion validity

We calculated bivariate correlations of the PTMQ scales with the MWMS scales, the PTI scale, and the TSE scale. Furthermore, a stepwise linear regression with forward selection of the PTMQ scales on PTI was calculated. In order to determine the incremental validity of the PTMQ over global work motivation, we also calculated bivariate correlations of the MWMs scales with PTI and TSE and conducted a stepwise linear regression with forward selection of the MWMS scales on PTI. In order to assess differences of the PTMQ scales dependent on lesson allocation, we first consolidated the two categories “I was asked whether I would like to take over teaching lessons” and “I was allocated without being asked” and compared their means against the category “I applied voluntarily for teaching” using t-tests for independent groups.

## Results

### Sample

Of the 645 distributed questionnaires, 247 were returned resulting in a response rate of 38.3 %. The characteristics of the sample are displayed in Table 1. Three participants answered less than 90 % of the PTMQ items, four participants of the MWMS items, two participants of the PTI items and nine participants of the TSE items and were therefore excluded from calculations involving the respective scale.

**Table 1:** Characteristics of the study sample

Age (Years)	Sex		Medical specialty		Teaching experience	Occupational position		Postdoctoral lecture qualification	
M (SD)					M (SD)/Years				
37.19 (7.83)	female	30.4 %	internal medicine	64.4 %	8.77 (7.46)	resident	50.8 %	yes	27.1 %
	male	69.6 %	surgery	35.2 %		consultant	12.3 %	no	72.5 %
			n/a	0.4 %		attending physician	33.6 %	n/a	0.4 %
						other	3.3 %		

### Factorial structure

**Fig. 1:**
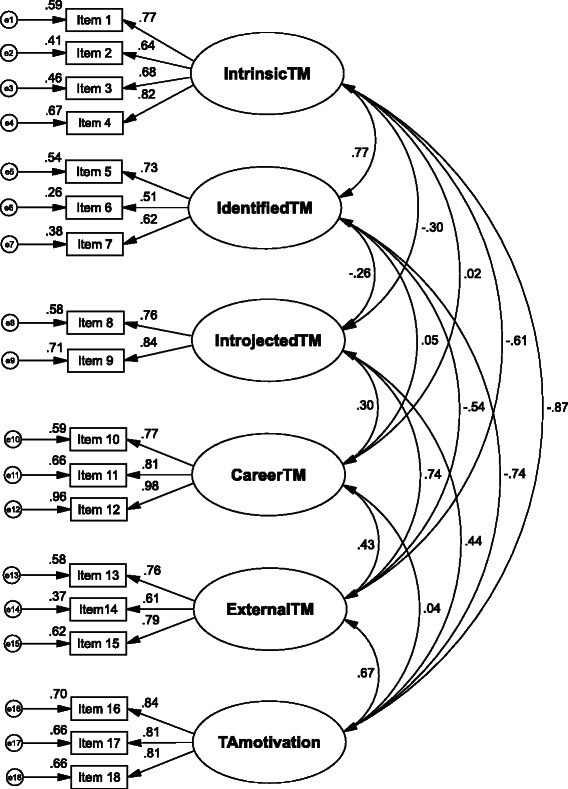
Factorial structure of the PTMQ: Factor loadings and indicator reliabilities

Following the structure of the MWMS, we primarily set up a model with the five main factors intrinsic teaching motivation (IntrinsicTM), identified teaching motivation (IdentifiedTM), introjected teaching motivation (IntrojectedTM), the first order factor external teaching motivation (ExternalTM_Model1) and teaching amotivation (TAmotivation). ExternalTM_Model1 comprised a second order factor for external-career teaching motivation (ExternalCareerTM_Model1) and a second order factor for other external items (ExternalOtherTM_Model1) including socially motivated teaching motivation. This resulted in four items for IntrinsicTM, three items for IdentifiedTM, two items for IntrojectedTM, and six items for ExternalTM_Model1 comprising three items for ExternalCareerTM_Model1 and ExternalOtherTM_Model1, respectively (Model 1). However, in this model ExternalCareerTM_Model1 showed a low factor loading of .37 on the first-order factor, while ExternalOtherTM_Model1 displayed a factor loading of 1.00 on the first-order factor. Therefore, we set up a second model with career teaching motivation (CareerTM, formerly ExternalCareerTM_Model1) as a distinct factor and ExternalTM (formerly ExternalOtherTM_Model1) as another distinct factor resulting in six first-order factors (Model 2, see Fig. 1).

As shown in Table 2, Model 2 shows better values for all global goodness-of-fit indicators compared to Model 1. Furthermore, as all global indicators suggest that it is consistent with the observed data, Model 2 was retained as the final structure of the PTMQ. The final items of the PTMQ are provided in Table 3.

**Table 2:** Global goodness-of-fit indicators for the two tested models

	TLI	RMSEA	SRMR	CFI
Model 1	.944	.057	.083	.954
Model 2	.957	.050	.055	.966

*TLI* Tucker-Lewis-index, *RMSEA* Root mean square error of approximation, *SRMR* Standardized root mean square residual, *CFI* Comparative fit index

**Table 3:** PTMQ items, means, standard deviations, skewness and kurtosis

Item		M (SD)	Skewness	Kurtosis
IntrinsicTM				
1	I look forward to my next teaching unit most of the time.	2.43 (0.86)	−0.28	−0.01
2	I enjoy my teaching most of the time.	3.11 (0.73)	−1.15	3.35
3	During teaching, I am completely in my element.	2.48 (0.94)	−0.35	−0.01
4	Teaching enriches my job.	2.79 (0.95)	−0.66	0.19
IdentifiedTM				
5	I teach because it’s important for me to make my contribution to students becoming good physicians in the future.	3.36 (0.79)	−1.29	1.69
6	I teach because I am convinced it’s a physician’s duty to pass on his knowledge.	3.22 (0.74)	−0.75	0.41
7	I teach because I find my lessons’ contents important.	3.07 (0.73)	−0.81	1.42
IntrojectedTM				
8	I teach because otherwise I would have a bad conscience towards my colleagues.	0.81 (0.83)	0.93	0.46
9	I teach because otherwise I would have a bad conscience towards my supervisors.	0.87 (0.89)	0.82	0.12
CareerTM				
10	I teach because I need the lessons to accomplish my occupational objectives.	1.72 (1.19)	0.11	−0.87
11	I teach because it is advantageous to my occupation.	1.88 (1.17)	−0.02	−0.89
12	I teach because it could promote my career.	1.67 (1.17)	0.13	−0.94
ExternalTM				
13	I teach most of the time because my supervisors expect it from me.	1.37 (1.03)	0.39	−0.44
14	I mainly teach because it belongs to my scope of duties.	2.00 (1.09)	−0.02	−0.72
15	I mainly teach because otherwise I would get into trouble with my supervisors.	0.89 (0.91)	1.02	0.88
TAmotivation				
16	I teach although teaching is rather irrelevant to me in comparison to my other occupational activities.	0.83 (0.89)	0.96	0.68
17	I teach although I hardly ever feel like doing it.	0.68 (0.83)	1.36	2.29
18	I teach although I often perceive it as an annoying chore.	0.83 (0.96)	1.17	1.11

*PTMQ* Physician Teaching Motivation Questionnaire; response range from 0 = “does not apply at all” to 4 = “fully applies”. The original items were developed in German language and tested in a German sample. English translations are provided for the readers’ convenience

When excluding CareerTM, all factors show their highest positive or least negative intercorrelations, respectively, with the factors closest to them in terms of amount of self-regulation, with decreasing positive or increasing negative intercorrelations, respectively, with factors more distinct in terms of self-regulation (Fig. 1). Deviating from this pattern, CareerTM only shows significant intercorrelations with its closest factors ExternalTM and IntrojectedTM, but no other significant intercorrelations. As for local goodness-of-fit, indicator reliabilities range from .26 until .96 with three items showing values <.40 (items 6 and 6 from the IdentifiedTM factor and item 14 from the ExternalTM factor; Fig. 1). All factor loadings were highly significant (*p* <.001).

### Scale characteristics

Table 4 presents the means, standard deviations, intercorrelations, and internal consistencies of the PTMQ scales. IdentifiedTM shows the highest means in our sample, followed by IntrinsicTM. IntrojectedTM and TAmotivation show the smallest means. All internal consistencies except for the IdentifiedTM scale are good as indicated by Cronbach’s α, while IdentifiedTM shows an acceptable internal consistency. The intercorrelations between the scales show the same pattern as the factor intercorrelations.

**Table 4:** Means, standard deviations, intercorrelations and internal consistencies of the PTMQ scales

Subscale	M	SD	1	2	3	4	5	6
1. IntrinsicTM	2.60	0.71	(.82)					
2. IdentifiedTM	3.23	0.57	.56**	(.65)				
3. IntrojectedTM	0.84	0.78	–.23**	–.19**	(.78)			
4. CareerTM	1.75	1.06	.04	.05	.24**	(.89)		
5. ExternalTM	1.42	0.83	–.49**	–.36**	.57**	.34**	(.75)	
6. TAmotivation	0.78	0.79	–.72**	–.55**	.38**	.04	.57**	(.86)

**p <.01 (two-tailed). Internal consistency values calculated as Cronbach’s α are presented diagonally in parentheses; *PTMQ* Physician Teaching Motivation Questionnaire, *TM* Teaching motivation, *T* Teaching

### Convergent validity with global work motivation

As shown in Table 5, IntrinsicTM, CareerTM and TAmotivation have their highest positive correlations with those scales of the MWMS which correspond in terms of content. IdentifiedTM shows the highest correlation with the intrinsic subscale of the MWMS, IntrojectedTM with the external-material subscale of the MWMS, and ExternalTM with the external-material subscale of the MWMS.

**Table 5:** Correlations of 1. the PTMQ scales with the MWMS scales, PTI and TSE and 2. the MWMS scales with PTI and TSE

1. PT MQ scales	IntrinsicTM	IdentifiedTM	IntrojectedTM	CareerTM	ExternalTM	TAmotivation
MWMS						
Intrinsic	.38^**^	.32^**^	–.16^*^	.02	–.30^**^	–.30^**^
Identified	.26^**^	.28^**^	.03	.04	–.05	–.16^*^
Introjected	.08	.10	.33^**^	.19^**^	.27^**^	.08
Extrinsic social	–.08	–.05	.33^**^	.32^**^	.41^**^	.15^*^
Extrinsic material	–.10	–.05	.36^**^	.34^**^	.41^**^	.18^**^
Amotivation	–.22^**^	–.14^*^	.05	.07	.17^**^	.25^**^
PTI	.54^**^	.51^**^	–.23^**^	.07	–.27^**^	–.46^**^
TSE	.42^**^	.36^**^	–.13^*^	.07	–.20^**^	–.31^**^
2. MWMS scales	MWMS intrinsic	MWMS identified	MWMS introjected	MWMS external material	MWMS external social	MWMS amotivation
PTI	.12	.28**	.11	–.10	.03	–.16*
TSE	.28**	.34**	–.01	–.14*	–.17*	–.11

*p <.05. **p <.01 (two-tailed). *PTMQ* Physician Teaching Motivation Questionnaire, *MWMS* Multidimensional work motivation scale, *PTI* Perceived teaching involvement, *TSE* Teaching self-efficacy, *TM* Teaching motivation, *T* Teaching

### Concurrent criterion validity

The more the subscales of the PTMQ represent a self-regulated type of motivation, the higher positive correlations they show with PTI. In contrast, IntrojectedTM, ExternalTM, and TAmotivation show higher negative correlations the less these subscales represent a self-regulated type of motivation as postulated by SDT with the exception of CareerTM, which shows no significant correlation with PTI. The same pattern can be found for TSE with smaller correlations. In contrast, the MWMS scales show either lower or non-significant correlations with both PTI and TSE.

A stepwise multiple regression of PTI on the PTMQ scales with forward selection shows significant positive β-coefficients for IntrinsicTM and IdentifiedTM (Table 6). These two variables together explain 34.8 % of the variance in PTI as derived from the corrected R^2^. A stepwise multiple regression of PTI on the MWMS scales with forward selection only shows a significant positive β-coefficient for the MWMS identified scale, explaining 8 % of the variance derived from the uncorrected R^2^.

**Table 6:** Stepwise multiple regressions with 1. PTI on the PTMQ scales and 2. PTI on the MWMS scales

Predictor	β	R	Adj. R^2^	ΔR^2^
*Step 1*		.540	.289	.292***
IntrinsicTM	.540***			
*Step 2*		.594	.348	.061***
IntrinsicTM	.374***			
IdentifiedTM	.298***			
MWMS identified	.283***	.080	.076	.080***

***p <.001 (two-tailed); only significant predictor variables are shown; forward selection; *PTMQ* Physician Teaching Motivation Questionnaire, *MWMS* Multidimensional work motivation scale, *PTI* Perceived teaching involvement

With respect to lessons’ allocation, physicians who stated to have primarily applied voluntarily for teaching in the year prior to this study show significantly higher values in IntrinsicTM and IdentifiedTM and significantly lower values in ExternalTM and TAmotivation as hypothesized (Table 7). They also show significantly lower values in IntrojectedTM, but no significant differences were found for CareerTM.

**Table 7:** T-tests of means in the PTMQ scales dependent on lesson allocation

PTMQ scale	Self-selection	Other selection	*t*-Test	
	M/SD	M/SD	t	*p*
IntrinsicTM	3.09 (0.57)	2.57 (0.69)	4.831	.000
IdentifiedTM	3.51 (0.50)	3.14 (0.57)	4.063	.000
IntrojectedTM	0.65 (0.68)	0.92 (0.78)	−2.177	.031
CareerTM	1.90 (1.11)	1.69 (1.04)	1.235	.218
ExternalTM	1.12 (0.58)	1.55 (0.87)	−4.059	.000
TAmotivation	0.39 (0.43)	0.90 (0.84)	−5.756	.000

*PTMQ* Physician Teaching Motivation Questionnaire, *TM* Teaching motivation, *T* Teaching

## Discussion

Since our initial literature search resulted in no validated instruments for the teaching motivation of hospital-based physicians, we developed and validated a new instrument, the Physician Teaching Motivation Questionnaire (PTMQ). It is based on the multidimensional conceptualization of motivation described by the self-determination theory [6]. The PTMQ’s factorial structure, its reliability, concurrent criterion validity and incremental validity support its suitability to assess physicians’ teaching motivation. Subsequently, we will discuss specific results und issues related to the PTMQ’s validation.

All global goodness-of-fit indicators suggest a good fit of the factorial structure of the PTMQ with the data. However, as item selection and the assessment of the fit indicators were conducted with the same sample and method, the factorial structure should be examined in further samples.

Cronbach’s alphas indicate good internal consistencies for all scales except for IdentifiedTM whose internal consistency must be denoted as “acceptable” following a standard classification recommendation [35]. However, our analyses of criterion validity indicate that the IdentifiedTM scale is nevertheless useful and in compliance with SDT. Also in compliance with SDT, bivariate correlations show positive and the second highest correlations with PTI and TSE, respectively, after IntrinsicTM. Furthermore, the regression analysis shows that IdentifiedTM adds incremental variance over IntrinsicTM to the explanation of PTI. The means of the PTMQ were highest for IdentifiedTM, followed by IntrinsicTM and lowest for IntrojectedTM and TAmotivation. This structure was also found for global work motivation measured by the MWMS in five of seven samples with different languages and countries [22]. Furthermore, the MWMS subscales show descending means depending on the degree of self-regulation of the subscale. With the exception of a low mean for IntrojectedTM and the highest mean for IdentifiedTM, we also found descending means from the higher self-regulated subscales to TAmotivation.

With respect to convergent criterion validity, all scales of the PTMQ show their highest or second highest correlations with the respective MWMS scales. As for criterion validity, they also show patterns of correlations with PTI and TSE that are consistent with SDT, with the exception of CareerTM. Furthermore, the type of lesson allocation is associated with the motivational dimensions as hypothesized. No association was found for CareerTM.

As an indicator of incremental validity, our results show that the PTMQ is far superior in predicting teaching involvement and that it shows higher and/or significant correlations with perceived teaching competence in comparison to global work motivation (MWMS). The fact that only two of our six teaching motivation scales contribute significantly to variance explanation in our criteria can be explained by the high intercorrelations of our factors. While the use of only seven items to predict teaching involvement is economic, this also raises the question how useful the other scales are and whether they are a necessary part of our newly developed instrument. This cannot be answered by our findings sufficiently. As other criterion variables could display considerable correlations, we recommend to use all scales. High scale intercorrelations were also found for measurements of global work motivation [25, 43]. However, in these validation papers, the instruments and their subscales were not validated using multiple regressions, so the results of our regression analysis cannot be compared.

While formulas have been suggested to calculate a relative autonomy index based on the substraction of values in the controlled and autonomous motivation subscales [44], little evidence for an underlying continuum structure was found in Rasch analyses [45]. Therefore, we do not recommend the consolidation of all subscales.

The PTMQ subscale CareerTM constitutes an exception in terms of its factor and scales intercorrelations as well as with respect to its criterion correlation patterns. Originally conceptualized as part of extrinsic teaching motivation and in consistence with this, it intercorrelates most highly with the ExternalTM factor of the PTMQ. However, our initial factor analysis indicated that it constitutes a separate factor. Furthermore, it shows no significant correlations with neither PTI nor TSE in contrast to the ExternalTM scale that correlates significantly negative with both criteria. An explanation for this deviation might be that career motivation is a multidimensional construct comprising underlying factors that include different degrees of intrinsic and extrinsic motivation, respectively. This assumption is supported by a study differentiating between intrinsic and extrinsic career motivation in which the number of publications of medical faculty members was positively associated with intrinsic career motivation and negatively associated with extrinsic career motivation [46]. Items for intrinsic career motivation included striving for personal challenge, increasing one’s knowledge and being given the opportunity to be creative and free of supervision, while extrinsic items included striving for money, social status and leadership. Therefore, it seems plausible that our items for CareerTM do not distinguish between intrinsic and extrinsic career motivations. The underlying antagonistic career motivations might neutralize each other, which could result in the missing relationships with other PTMQ scales as well as with PTI, TSE and lessons’ allocation. Another explanation for the deviating scale characteristics of CareerTM might be that career in our sample was not perceived as a motivating factor [35] as at German Medical Faculties in University Hospitals all residents are involved in clinical teaching independently of their intended career paths. Therefore, the intercorrelations between CareerTM, PTI, TSE and lesson allocation might be different in populations or academic cultures where financial or career-related incentives with respect to teaching are in effect.

The PTMQ might be a suitable instrument for adding an additional teacher-centred dimension to the evaluation of teaching in a clinical context as proposed by Ten Cate et al. [21]. It could be used especially for the evaluation of quality-ensuring and -enhancing measures such as new learning formats, new curricula, teacher trainings and other means of faculty development. Low levels of the autonomous subscales IntrojectedTM and IdentifiedTM and high levels of ExternalTM and TAmotivation will indicate deficits in the motivational status and detect the necessity to develop self-regulation-enhancing measures, which target the satisfaction of the basic needs as proposed by SDT, autonomy, perceived competence and relatedness. While the causality has yet to be clarified, examples of such measures related to basic need satisfaction were suggested by Engbers et al. [47]. Even if these or other measures might not immediately affect student evaluations, empirical findings show that this will increase the well-being of employees [6], which is an important factor to prevent issues such as burn-out [48]. Apart from the practical use of our questionnaire, we suggest to use it in research that is targeted at developing and evaluating autonomy-enhancing measures.

### Limitations of this study

One important limitation of this study is that all data were collected cross-sectionally by means of self-reports which bears the risk of common method variance (CMV) issues. These can arise when participants of a study present the source for the independent and the dependent variables at the same time. While some authors argue that CMV issues are exaggerated [49], Richardson et al. found in a simulation study that even in the presence of CMV, “the absolute correction accuracy of all (control) techniques tended to be low“[50]. Apart from this, the fact that several of the correlations of the MWMS scales with PTI and TSE in our study were not significant indicates that our significant findings are less likely to be artefacts [50]. In general, there is good reason to assume that the subjectivity of our criteria due to self-report might be a smaller issue for TSE than PTI. Self-efficacy has been conceptualized as an explicitly subjective construct and numerous studies, including academic and general occupational contexts, prove its predictive value for performance as an outcome of motivation, independent from objective measures of competence [51–57].

Furthermore, our PTI and TSE measuring instruments have not been validated beforehand. However, our literature search yielded no validated instruments measuring PTI and TSE in the field of medical education. It can be assumed that instruments aimed at measuring work-related global constructs such as commitment, turnover intention, emotional exhaustion or job effort are not suitable for relationship measurements with respect to specific occupational tasks like teaching. The low or non-significant correlations, respectively, of the MWMS scales – although the MWMS has been validated profoundly across several countries – with the PTMQ underscore the necessity of instruments tailored to physicians’ teaching activities. Even though great effort and care has been put in the development of all scales, our interpretations about the concurrent validity with respect to PTI and PTC have to be looked at with great caution.

In this study, the sample consisted of physicians from internal medicine and surgery only. However, the PTMQ has been designed for hospital-based physicians in general and should be suitable for other specialties as well. Furthermore, while the sample included physicians who teach students, there are no known theoretical obstacles to applying the PTMQ to teachers teaching residents and fellows. Our response rate of 38.3 % might raise some doubt about potentially biased results. Surveys with physicians generally tend to have lower response rates than surveys with other populations [58]. Furthermore, questionnaires for physicians with more than 1000 words had lower response rates (38.0 %) than those below 1000 words (59.4 %) [59]. Our questionnaire included 2520 words. Response rates varied from e.g. 13.3 % of surgeons in one hospital to 78.9 % of internists another hospital. Samples with low response rates might be assumed to show higher degrees of autonomous and lower degrees of controlled motivation in comparison to their populations. Hence, it seems more likely that physicians with less autonomous teaching motivation did not respond. However, we found no correlations between response rates and any of our motivational dimensions. Nevertheless, our data should be interpreted with caution. With respect to the factorial structure of our questionnaire, no potential bias seems evident due to the response rate.

### Future research

One advantage of using the same instrument consistently across different institutions, departments and countries lies in the comparability of the results. Therefore, it is generally beneficial to establish instruments, which have been validated for different research questions. Hence, translations of the PTMQ into other languages and corresponding validations seem promising. Further research is also needed to determine the research questions and the study designs for which the PTMQ is suitable. For example, in order to determine the PTMQ’s suitability for studies with a pre-post-design such as evaluations of faculty development programs, teacher trainings and other interventions, future studies should investigate the instrument’s sensitivity to change.

In this study, teaching involvement was measured using a self-rating instrument. In future studies, more objective data on the impact of teaching motivation on teaching involvement should be gathered. Furthermore, it should be investigated whether the PTMQ can predict teaching quality, students’ learning motivation and, as a consequence, students’ learning advances. Additionally, the characteristics of the CareerTM scale and its associations with different outcomes in different samples should be further explored. If future studies in other settings should also find that this scale shows no significant associations with relevant criteria, a modification of this scale would be necessary. In such a case, we would recommend to develop items, which distinguish between intrinsic and extrinsic career motivations with respect to teaching. Most modern learning formats as well as curriculum and faculty development programs are student-centered in conception and focus on student-centered outcomes when being evaluated. Ten Cate et al. point out that this one-sided perspective might cause conflicts with physicians’ teaching motivations [21]. Therefore, future studies should explore both perspectives, identify potential areas of conflict and provide solutions for them.

## Conclusions

Assessments of factorial validity, internal consistency, concurrent convergent validity and incremental validity indicate generally a good usability of the PTMQ as an instrument to measure teaching motivation in hospital-based physicians.

### Ethics

The study was performed in accordance with the Declaration of Helsinki and the Ethics Committee of the Chamber of Physicians, Hamburg, confirmed the innocuousness of the study protocol.
